# Haemoptysis Caused by Right Intercostal Artery‐To‐Pulmonary Artery Fistulas Mimicking Cryptogenic Haemoptysis

**DOI:** 10.1002/rcr2.70587

**Published:** 2026-04-22

**Authors:** Kengo Sato, Masahiro Kawashima, Masaomi Maeda, Ibuki Kosai, Takafumi Kato, Keita Takeda, Hiroshi Igei, Kimihiko Masuda, Yoshiteru Morio, Hirotoshi Matsui

**Affiliations:** ^1^ Center for Pulmonary Circulation and Hemoptysis National Hospital Organization Tokyo Hospital Kiyose Japan

**Keywords:** embolisation, fistulas, haemoptysis, intercostal artery, pulmonary artery

## Abstract

Systemic artery‐to‐pulmonary artery fistulas (SA‐PAFs) are rare vascular anomalies, typically associated with chronic pulmonary or pleural inflammation. SA‐PAFs are categorised by their systemic arterial origin, arising either from the bronchial arteries or nonbronchial systemic arteries such as the intercostal arteries. Nonbronchial SA‐PAFs are uncommon in patients without a history of thoracic trauma, surgery or infection. We report a 55‐year‐old man with recurrent haemoptysis caused by right intercostal artery‐to‐pulmonary artery fistulas, despite having no history of pulmonary or pleural disease. The diagnosis was established by selective angiography, and the lesions were successfully treated with coil embolisation. This case demonstrates that haemoptysis due to nonbronchial SA‐PAFs can occur even in the absence of a history of the usual aetiologies and may be misclassified as cryptogenic haemoptysis. Even in patients initially diagnosed with cryptogenic haemoptysis, it is crucial to first perform a detailed vascular evaluation using CT angiography to identify hidden nonbronchial SA‐PAFs.

## Graphical abstract

We report a rare case of haemoptysis caused by systemic artery‐to‐pulmonary artery fistulas (SA‐PAFs) originating from the right intercostal arteries in a patient without underlying pulmonary disease. This case highlights the diagnostic importance of careful evaluation with CTA and angiography, especially in patients initially diagnosed with cryptogenic haemoptysis.

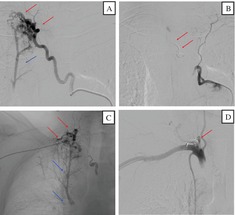

## 1 Introduction

Systemic artery‐to‐pulmonary artery fistulas (SA‐PAFs) are rare vascular anomalies characterised by abnormal communications between systemic arteries and the pulmonary artery (PA). Unlike systemic‐to‐pulmonary shunts mediated by a microvascular capillary network, SA‐PAFs represent direct, macroscopic communications between systemic and pulmonary arteries. While patients with SA‐PAFs are typically asymptomatic, they may occasionally present with haemoptysis [1]. The bronchial arteries are the primary source of haemoptysis, whereas nonbronchial systemic arterial sources are relatively infrequent, particularly in patients without underlying pulmonary or pleural disease [2]. Importantly, in the absence of a clear clinical history of pulmonary or pleural disease, SA‐PAFs arising from nonbronchial systemic arteries may be overlooked on initial evaluation. This can result in a misdiagnosis of cryptogenic haemoptysis. We report a rare case of recurrent haemoptysis in a patient with no definitive history of pulmonary or pleural disease. Although the patient was initially diagnosed with cryptogenic haemoptysis, the bleeding was subsequently attributed to right intercostal artery‐to‐pulmonary artery fistulas and was successfully managed with transcatheter coil embolisation.

## 2 Case Report

A 55‐year‐old man was initially diagnosed with cryptogenic haemoptysis, which was managed with haemostatic agents. Three years later, he presented to our hospital following a recurrence of haemoptysis.

**FIGURE 1:**
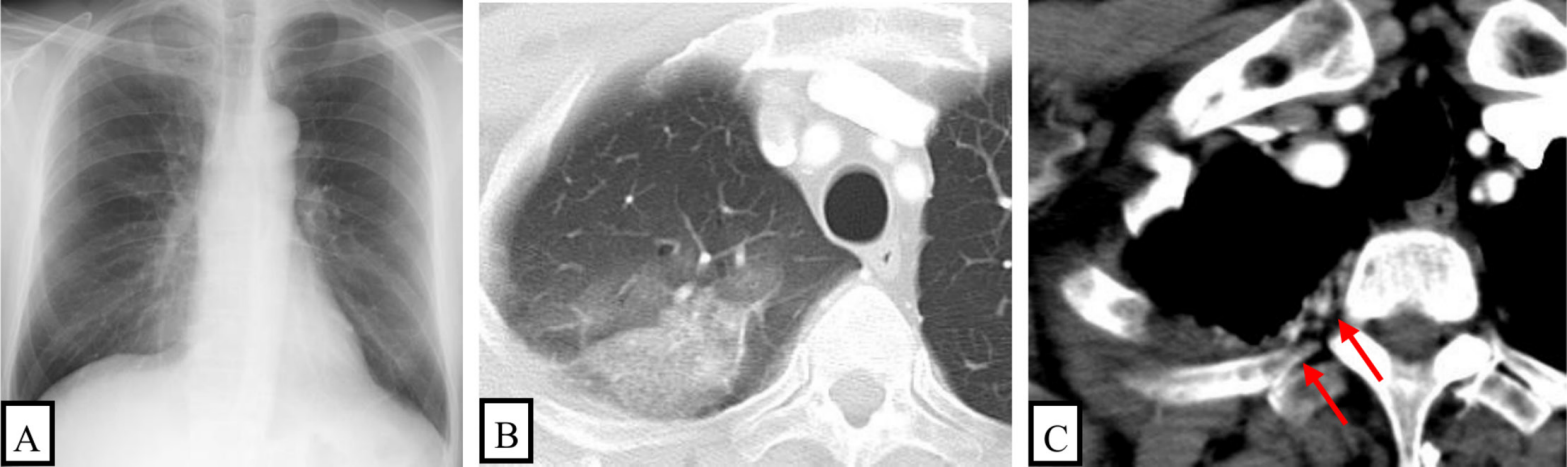
(A) Chest radiography showed decreased lucency in the right lung apex. (B) Axial images from CTA showed ground‐glass opacities and consolidation in the right upper lobe, consistent with aspirated blood. (C) Focal pleural thickening (3 mm) with enhancing vascular structures (red arrow) was observed in the right lung apex.

**FIGURE 2:**
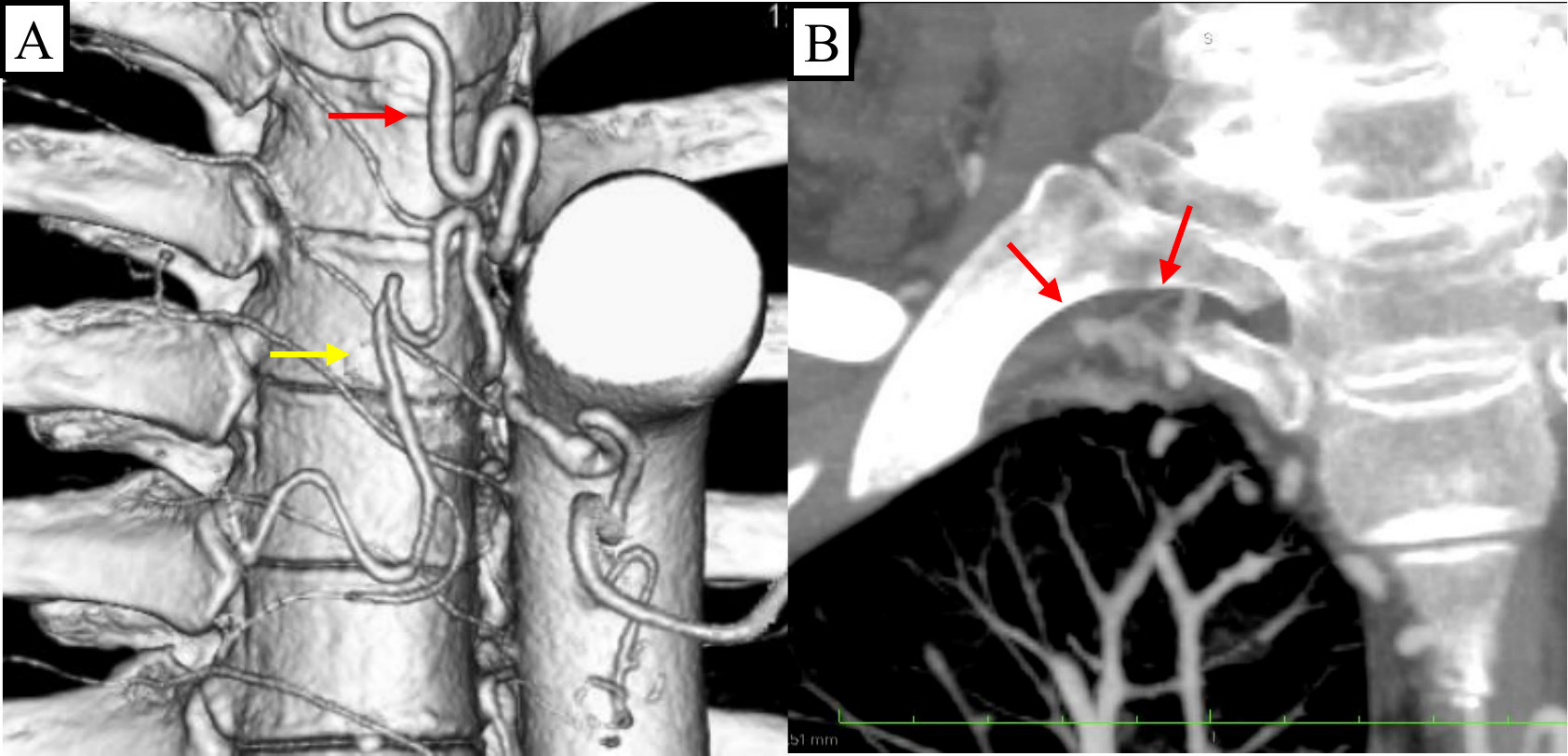
(A) Three‐dimensional CTA demonstrated mild dilation of the right bronchial artery (2.1 mm at the ostium) (yellow arrow) and marked dilation and tortuosity of the right second intercostal artery (2.5 mm) (red arrow). (B) Coronal images from CTA showed marked dilation and tortuosity of the right supreme intercostal artery (2.7 mm) (red arrow).

He had no history of thoracic trauma, surgery, pulmonary infection or congenital heart disease and had a 32‐pack‐year smoking history. Vital signs, physical examination and laboratory studies were unremarkable. Microbiological studies were negative. Chest radiography showed decreased lucency in the right lung apex (Figure 1A). Computed tomography angiography (CTA) was performed using bolus tracking with a region of interest placed in the descending aorta, with image acquisition commencing 8–10 s after the attenuation threshold was reached. CTA demonstrated ground‐glass opacities and consolidation in the right upper lobe, consistent with aspirated blood (Figure 1B). Focal pleural thickening (3 mm) with enhancing vascular structures was observed in the right lung apex (Figure 1C). There was no evidence of underlying parenchymal disease. CTA also demonstrated the following (Figure 2): (1) mild dilation of the right bronchial artery (2.1 mm at the ostium); (2) marked dilation and tortuosity of the right supreme intercostal artery (2.7 mm); and (3) marked dilation and tortuosity of the right second intercostal artery (2.5 mm). These arteries were suspected to be the bleeding sources.

**FIGURE 3:**
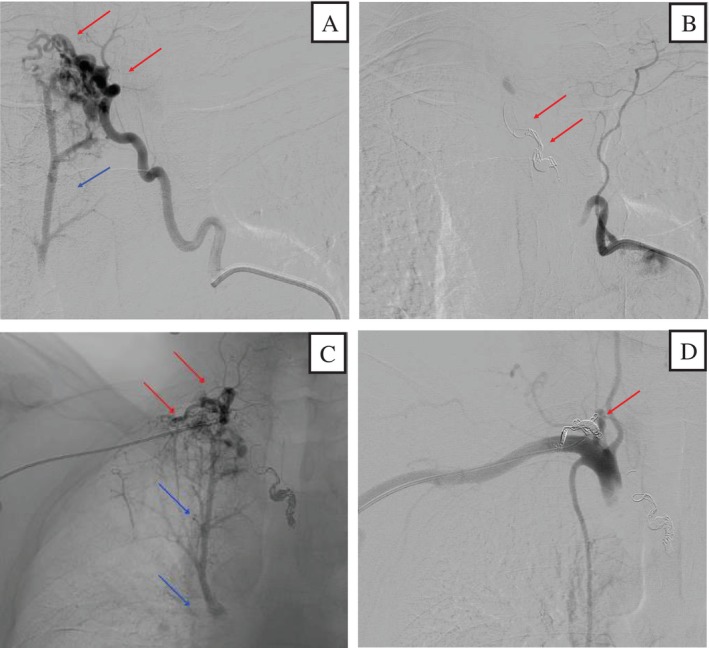
(A) Selective angiography of the right second intercostal artery revealed marked tortuosity, microaneurysm formation (red arrow) and rapid, direct opacification of the pulmonary artery without an intervening capillary phase, consistent with a direct PA fistula (blue arrow). (B) Post‐embolisation angiography confirmed successful occlusion of the right second intercostal artery (red arrow) and disappearance of the direct PA fistula. (C) Selective angiography of the right supreme intercostal artery showed tortuous dilation with abnormal neovascularisation (red arrow) and direct opacification of the pulmonary artery without a capillary phase, also consistent with a direct PA fistula (blue arrow). (D) Post‐embolisation angiography of the right supreme intercostal artery showed complete occlusion of the vessel (red arrow) and disappearance of the direct PA fistula.

Transcatheter coil embolisation was performed in two sessions. In the first session, via a transfemoral approach, selective angiography of the right intercostobronchial trunk demonstrated only mild dilation of the bronchial artery, without evidence of a PA shunt or parenchymal blush in the right upper lobe. It was therefore not considered the bleeding source. In contrast, selective angiography of the right second intercostal artery revealed marked tortuosity, microaneurysm formation and rapid, direct opacification of the PA without an intervening capillary phase, consistent with a direct PA fistula (Figure 3A). The lesion was treated with coil embolisation (Figure 3B). In the second session, via a transradial approach, selective angiography of the right supreme intercostal artery showed tortuous dilation with abnormal neovascularisation and direct opacification of the PA without a capillary phase, also consistent with a direct PA fistula (Figure 3C). Coil embolisation was successfully performed (Figure 3D). The patient has had no recurrence of haemoptysis for more than 10 years since discharge.

## 3 Discussion

This case highlights two key clinical lessons. First, although SA‐PAFs are typically associated with chronic pulmonary or pleural inflammation, they can occur in patients without a definitive history of pulmonary or pleural disease. Second, nonbronchial SA‐PAFs may be overlooked on initial evaluation, potentially leading to a misclassification as cryptogenic haemoptysis.

Pulmonary vascular anomalies are classified as congenital or acquired. Acquired SA‐PAFs are more common and are typically associated with thoracic trauma, surgery, malignancy or infection [1]. Nonbronchial systemic arteries have traditionally been reported to account for approximately 5% of haemoptysis sources [3], though recent reports from high‐volume centres suggest substantially higher rates, ranging from approximately 20% to 66% [4, 5]. Although haemoptysis from nonbronchial SA‐PAFs remains exceedingly rare in the absence of underlying pulmonary disease, as in the present case, these lesions should not be dismissed as a mere rarity.

In this patient, CT showed no evidence of active lung disease, such as bronchiectasis. However, focal pleural thickening was present and could have been easily overlooked. Chronic pleural inflammation is known to promote collateral vessel formation from systemic arteries, including the internal thoracic, intercostal, subclavian, axillary and inferior phrenic arteries, which may lead to SA‐PAF formation [1, 2, 6]. Nonetheless, SA‐PAFs associated with subtle pleural changes in the absence of overt chronic inflammatory lung disease remain underrecognised. Although a congenital vascular anomaly cannot be completely excluded, the overall clinical imaging features in this case are more consistent with an acquired SA‐PAF arising from mild pleural changes. This patient had no definitive history of thoracic trauma or respiratory infection. However, CT revealed focal pleural thickening. This feature likely represents a sequela of an unrecognised infection, such as pneumonia or pleurisy. Recognising focal pleural thickening can thus provide a critical diagnostic clue in identifying the source of haemoptysis, even without a clear medical history.

Approximately 20% of haemoptysis cases are classified as cryptogenic, often characterised by bronchial artery abnormalities, and typically respond well to bronchial artery embolisation [7]. However, as illustrated in this case, SA‐PAFs may be overlooked in patients diagnosed with cryptogenic haemoptysis. Pursuing a definitive aetiology through CTA and angiography is essential to identify treatable vascular lesions.

In our case, the initial evaluation failed to identify a bleeding source, leading to a diagnosis of cryptogenic haemoptysis. However, recurrent bleeding prompted further imaging, revealing the SA‐PAFs originating from the right intercostal arteries and identifying the culprit vessels suitable for transcatheter embolisation. This case underscores that careful imaging evaluation can uncover a treatable nonbronchial SA‐PAF in patients with haemoptysis initially labelled cryptogenic.

In summary, haemoptysis caused by nonbronchial SA‐PAFs can occur even without a clear medical history of pulmonary or pleural disease and may be misdiagnosed as cryptogenic. Even in patients initially diagnosed with cryptogenic haemoptysis, it is crucial to first perform a detailed vascular evaluation using CTA to identify hidden nonbronchial SA‐PAFs.

## Author Contributions

Kengo Sato drafted the manuscript and conducted the literature review. Masahiro Kawashima, Masaomi Maeda, Ibuki Kosai, Takafumi Kato, Keita Takeda, Hiroshi Igei, Kimihiko Masuda, Yoshiteru Morio and Hirotoshi Matsui reviewed and provided critical feedback on the manuscript. All authors read and approved the final version of the manuscript.

## Funding

The authors have nothing to report.

## Consent

The authors declare that written informed consent was obtained for the publication of this manuscript and accompanying images and attest that the form used to obtain consent from the patient complies with the Journal requirements as outlined in the author guidelines.

## Conflicts of Interest

The authors declare no conflicts of interest.

## Data Availability

The data that support the findings of this study are available from the corresponding author upon reasonable request.
